# Presidential Address

**Published:** 1886-12

**Authors:** F. Poole Lansdown

**Affiliations:** Senior Surgeon to the Bristol General Hospital


THE BRISTOL

### Medico-Chirurgical Journal.

DECEMBER, 1886.

### PRESIDENTIAL ADDRESS.

Delivered before the Bristol Medico-Chirurgical Society,

SESSION 1886-7.

BY

F. POOLE LANSDOWN, M.R.C.S.,

*Senior Surgeon to the Bristol General Hospital.*

GENTLEMEN,

Before proceeding to give you the few thoughts which have occurred to me as suitable to the present occasion, I must tender you my best thanks for electing me to this honourable position. As I told you, last October, I felt considerable diffidence in accepting the proffered honour; so now I feel not a little disturbed at the prospect of following so many distinguished friends who have so ably filled this chair. I can make no pretension to emulate them in their learned addresses; but I can assure you that it will be my earnest endeavour to discharge the duties of the office to the best of my ability, with due regard to its dignity and efficiency; and I feel that in this endeavour I shall be supported by your sympathy and forbearance for the shortcomings of a man who makes no pretension to superior wisdom or knowledge, but simply aims at doing his duty with the feeble powers that God has endowed him.

I purpose occupying your time very briefly by a few remarks on the progress of practical surgery during my professional career. It is now nearly thirty years, since being qualified, I have been engaged in hospital work; and although it has fallen to my lot to perform many hundreds of operations, I regret to say that I have no continuous record of my work: and the only excuse I can make for this grievous loss is the usual one, of want of time; for I have always found that each day in a busy life brings with it its own work, and notes which have been put off for a more convenient season have never been accomplished. On this account, I have no data to fall back upon for any special subject.

It has often been said, and I think with reason, that much of the improvement in modern surgery is due to the increase of specialisms; and also, I would add, in great measure by the influence of societies, such as our Medico-Chirurgical Society, in diffusing the knowledge acquired by the research and practice of such specialists. Now, when I come to think what vast strides practical surgery has made during my recollection, I feel considerable embarrassment as to what points I should direct your attention.

It seems to me that in the first place we must look to anæsthetics as the starting point of all the improvements in operative surgery. There are, doubtless, some of my hearers present who remember the horrors of the operating theatre in the pre-anæsthetic period. I have a lively recollection of it myself; for, as a schoolboy, my father used to take me to many of his operations—in order, I suppose, to give me a taste for my future profession; and I must confess that I think it had that effect, for I have always been more at home with the practical work rather than with the theoretical. At that time, brilliancy and speed were the great aims of the surgeon, in order to get his patient over the painful ordeal with as much dispatch as possible. Now all that is changed, and we are able deliberately to carry out our plans, with greater precision and more accuracy, without inflicting any pain. And here I would lay special claim to the value of chloroform, notwithstanding the way in which it is attempted to be discredited (especially in the pages of the *British Medical Journal*), some people going so far as to say that a surgeon using it ought to be indicted for manslaughter in case of fatal result. I maintain that it is the best and safest anæsthetic we possess, when carefully administered, and this by the simplest face-piece, with sponge in the centre, such as I have always used. Of course, we cannot forget that there is a certain amount of risk attending its use, by its supposed inhibitory effect on the nervous centres of the heart; but this is far outbalanced, in my opinion, by the blessings to the patient, for which the risk is worth incurring. We all know how in prolonged operations there is often considerable shock attending its administration; but then we must consider how many patients formerly died from shock alone, and some even from fear before the operation. I have known puerperal cases under its influence for fourteen and sixteen hours without any ill effect whatever. Æther is looked upon by many as the only safe anæsthetic. I differ from them. I remember the day when chloroform took its place, and with what joy

æther was discarded, with all the cumbersome apparatus necessary for its administration, the disagreeable taste attending its use permeating the breath for hours. Its more speedy effect in producing anæsthesia, and the facility of its administration, have been the reasons why chloroform has held its ground so long. I have never used any other compound, excepting nitrous oxide gas at the dentist's; and, personally, I have never had cause to regret my choice.

I should say that the development of hæmostatics was the next cause for the great advance in practical surgery. In my early days, silk ligatures were solely employed to tie the vessels, ulceration of the artery and suppuration in the deep parts of the wound being the necessary consequence of their removal, thus materially retarding the healing process. Torsion was introduced with great advantage. Then acupressure came into vogue: this was put aside altogether when research brought us the catgut ligature, which has now been so perfected that nothing better can be desired than chromicized catgut and Spencer Wells' forceps for staying all bleeding in wounds. In this connection I must refer to Esmarch's bandage and tourniquet, by which what is called the bloodless method of operating has been such a boon. It facilitates the exploration of diseased joints, and saves much loss of blood in the removal of sequestra from necrosed bones; and no doubt it has considerably reduced the mortality in amputations. It is true that objection is sometimes made to its use, from the amount of oozing consequent upon its removal; but this inconvenience can be remedied by first tying all visible vessels, then elevating the stump, and by using pressure with pad and bandage.

Then I would say that the antiseptic system of Sir J. Lister has done more in advancing surgical science than anything else. What untold advantages have followed its use in mitigating the sufferings and risks of operations! How rarely do we now see erysipelas, or hospital gangrene, which used to be the bane of our surgical wards—in many cases leading to the suggestion and use of separate houses, and even tents, for the better treatment of operation cases! The system so ably invented and perfected by Sir Joseph Lister leaves nothing to be desired. Faith in his system has led to the impunity with which the peritoneal cavity can now be dealt with; and the vast strides made in abdominal surgery are the outcome of this system. And yet many surgeons have now discarded the use of carbolic spray. Mr. Lawson Tait goes so far as to say that he would dress his wounds with pads of germs if he could collect a sufficiency for the purpose; and he tells us that he washes out the peritoneal cavity with ordinary company's water teeming with animalculæ. This seems rather heroic language; and one would be inclined to say that there is some truth in the old adage, "Familiarity breeds contempt." And yet what marvellous results he obtains! I remember at one time I came to the determination never to operate again on cases of cancer of the breast with secondary infiltration of the axillary glands, because I had lost a patient from septicæmia following this operation: but when I became familiar with the Listerian system, my fears were dispelled; and now I do not hesitate to recommend the operation, but advocate it most strongly—feeling secure in the preservative action of carbolic acid, and the good effects of perfect drainage, as the sure means of prolonging life in this dire disease.

I saw a patient last month for whom I removed a very bad scirrhus of the breast, with infiltrated axillary glands, *some six years ago;* and she is now in perfect health, with a clean cicatrix. And this is only one of many who have had their lives prolonged by timely removal of the disease. Doubtless you are all familiar with many such cases. One can hardly realise what great triumphs have been brought about by all these modern improvements. In reflecting, I have often been struck by the fact that amputations are not so numerous as they were formerly; and this is due, I believe, to the teaching of such men as Mr. Hilton, in his lectures on the Influence of Rest, and Sir Wm. Ferguson, who did so much, in those so-called days of conservative surgery, in advancing the treatment of joint disease. In those days, nearly every joint in the body was excised, thereby saving limbs which before were sacrificed; but since the introduction of antiseptics, and the confidence given to us by the almost certain absence of risk of inflammation in opening joints under the spray, we have found that incision, with clearing out of *débris* and diseased membranes, together with drainage and proper care, has taken the place of excision, which is now almost as rare an operation as amputation. And we are not only able to save the limbs, but in many cases to ensure a movable and useful joint, where formerly anchylosis was looked upon as the most favourable result. Then, again, who can doubt but that operations are far more numerous than they used to be? And things are now accomplished which would not have been attempted by our forefathers. Look, for instance, to what perfection abdominal surgery is brought: kidneys, spleen, entire uterus and its appendages, are removed—the latter being frequently removed with impunity and advantage to many sufferers; explorations of liver and gall bladder. Then, again, have not the larynx and œsophagus and large tracts of intestine been removed with safety? And ovariotomy—to what perfection that has been brought by the untiring energy of research!

Mr. Erichsen, in his address at Brighton, says there is no doubt we have reached finality in the mere mechanical art of surgery, both as to the expansion of its limits and the precision of its practice; but that we are yet on the threshold of the science of surgery: and so far as the treatment of wounds is concerned, almost absolute precision of result has been reached by the employment of strict hygienic precautions, and the adoption of the antiseptic method. The surgeon has thus arrived at this point of perfection—that he can assure his patient that there will be no suffering during the operation, as this is guarded against by anæsthetics; and no pain afterwards, for that can be averted by antiseptic dressings. We now know that the wound itself is not painful, but that after-pain is the direct result of septic inflammation: if this be prevented, and if healing by direct union be obtained —as it may be with almost absolute certainty under the improved dressings—no after-pain will result.

We see surgical science advancing every day. You have only to look at the report of cases by Mr. Victor Horsley, in last week's *Journal*, as evidence of the great advance in brain surgery, owing to the precision of diagnosis following in the path of researches on the localisation of cerebral disease by Ferrier and others. And it is due to antiseptics, aided by the skill of the operator, that such marvellous results can be obtained; for no surgeon would dare to cut away portions of the brain without faith in the preservative effect of such agents. What may be in store for the future generations by the use of electricity, no one can tell. Already it helps us: by its lighting power, as in its adaptation to the ophthalmoscope and other instruments for examining the cavities; by its heat, in the electric cautery, and by its chemical or disintegrating power on the blood and tissues; by electrolysis, in dispersing nævi and other tumours; and also in the treatment of strictures.

I must now bring these remarks to a close, and apologise to you for the very discursive and crude manner in which I have brought them to your notice.

